# Recurrent Gliosarcoma in Pregnancy

**DOI:** 10.1155/2014/953184

**Published:** 2014-05-12

**Authors:** İsmail Gülşen, Hakan Ak, Tevfik Yilmaz, Mehmet Deniz Bulut, İsmet Alkış, İrfan Bayram

**Affiliations:** ^1^Department of Neurosurgery, School of Medicine, Yüzüncü Yıl University, Van, Turkey; ^2^Department of Neurosurgery, School of Medicine, Bozok University, Yozgat, Turkey; ^3^Department of Neurosurgery, School of Medicine, Dicle University, Diyarbakır, Turkey; ^4^Department of Radiology, School of Medicine, Yüzüncü Yıl University, Van, Turkey; ^5^Department of Obstetrics and Gynecology, School of Medicine, Yüzüncü Yıl University, Van, Turkey; ^6^Department of Pathology, School of Medicine, Yüzüncü Yıl University, Van, Turkey

## Abstract

Gliosarcoma is a rare tumor of the central nervous system and it constitutes about 1 to 8% of all malignant gliomas. In this report we are presenting a recurrent gliosarcoma case during a pregnancy in a 30-year-old woman. This is the first report presenting gliosarcoma in the pregnancy.

## 1. Introduction

Gliosarcoma is a rare primary tumor of the central nervous system and it contains malignant glial and sarcomatous components [1, 2]. It constitutes about 1 to 8% of all malignant gliomas. The literature constitutes retrospective case series and case reports [2]. It is more commonly seen during fifth to seventh decades of life and predominantly in male gender [3].

The clinical symptoms and signs of gliosarcoma are similar to other space occupying lesions in the brain. Symptoms related with increased intracranial pressure like headache, vomiting, alterations in consciousness, and third and sixth nerve palsies are frequently seen. Headache, epileptic seizure, and hemiparesis are seen in about more than half of the cases [4]. The management of gliosarcoma consists of surgery, postoperative radiotherapy, and chemotherapy. However, there is no data in the literature about their management during pregnancy.

Herein, we are reporting a gliosarcoma case that diagnosed and operated three times during pregnancy.

## 2. Case

**Figure 1:**
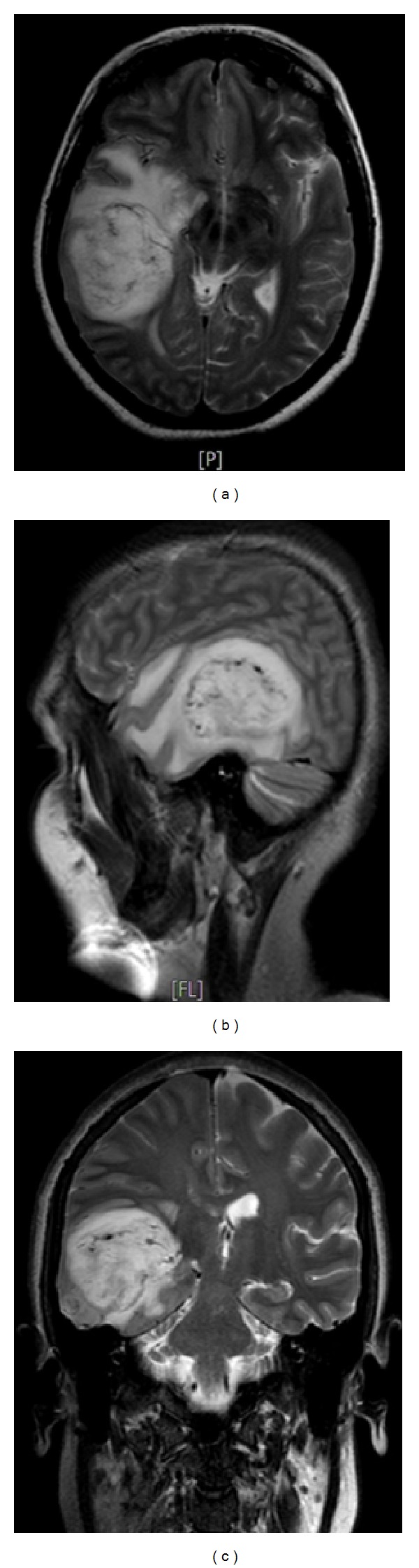
T2 weighted MR images on axial (a), sagittal (b), and coronal planes (c) show heterogenous hyperintense mass lesion on right temporooccipital lobe with prominent peripheral vasogenic edema.

**Figure 2:**
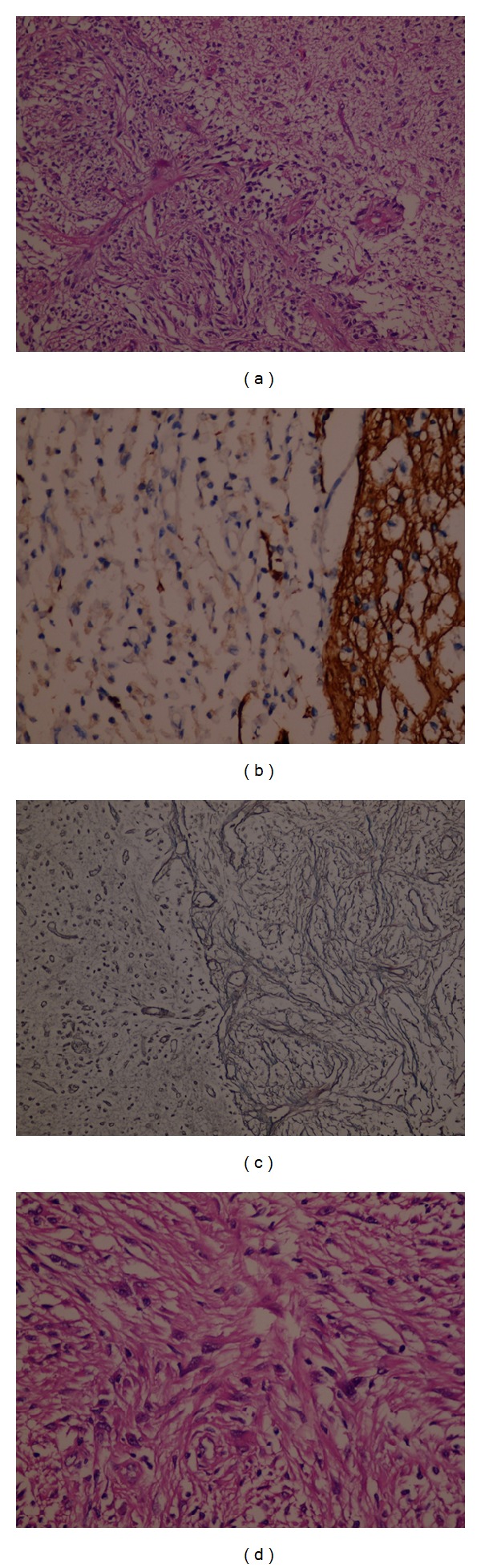
Pathological images of the lesion (a) gliosarcoma. A biphasic tissue pattern with areas displaying gliomatous and mesenchymal differentiation (H&E stain, original magnification ×200). (b) The gliomatous component shows strong GFAP expression and is separated from mesenchymal tumour cells without GFAP expression (immunoperoxidase stain, original magnification ×400). (c) Dense reticulin network shows that the sarcomatous area is well demarcated from the glial tumour portion (reticulin stain, original magnification ×200). (d) Sarcomatous area consisting of spindled and pleomorphic cells arranged in a storiform pattern (H&E stain, original magnification ×400).

**Figure 3:**
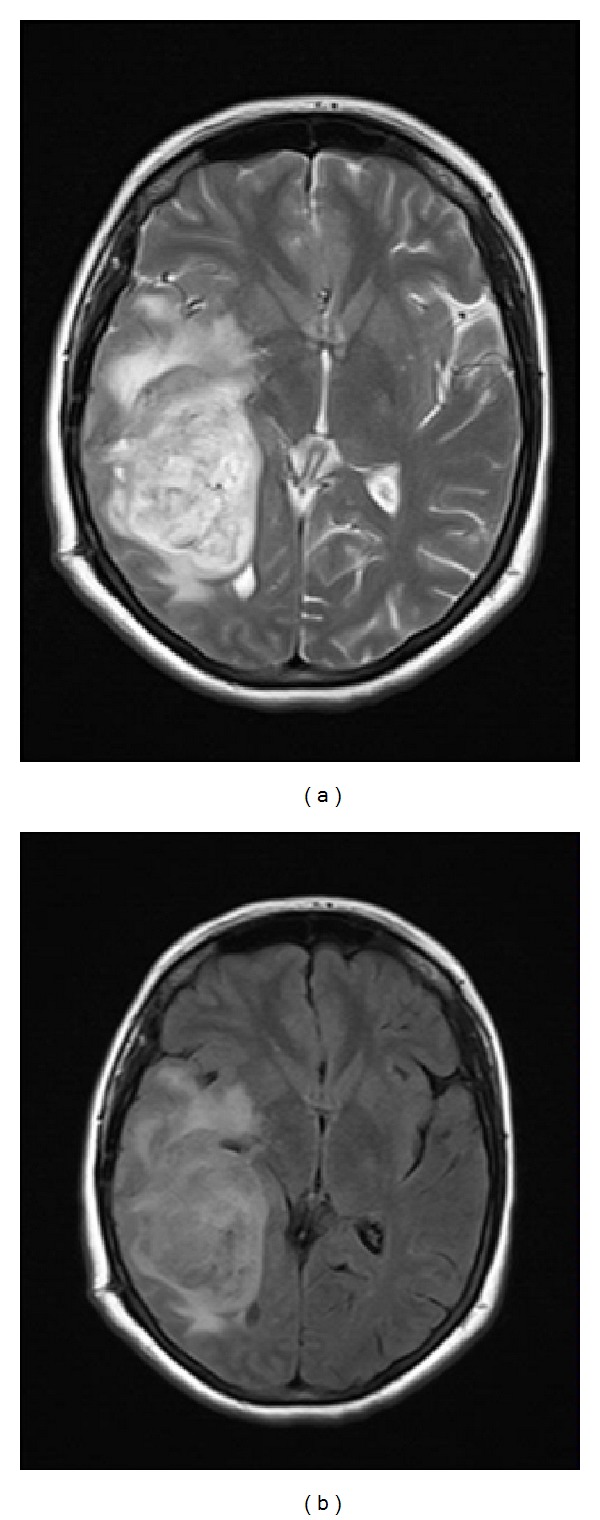
T2 weighted (a) and (b) FLAIR MR images of the lesion before second operation on axial plane showing a recurrent lesion with prominent peripheral edema and minimal midline shift at the same location.

**Figure 4:**
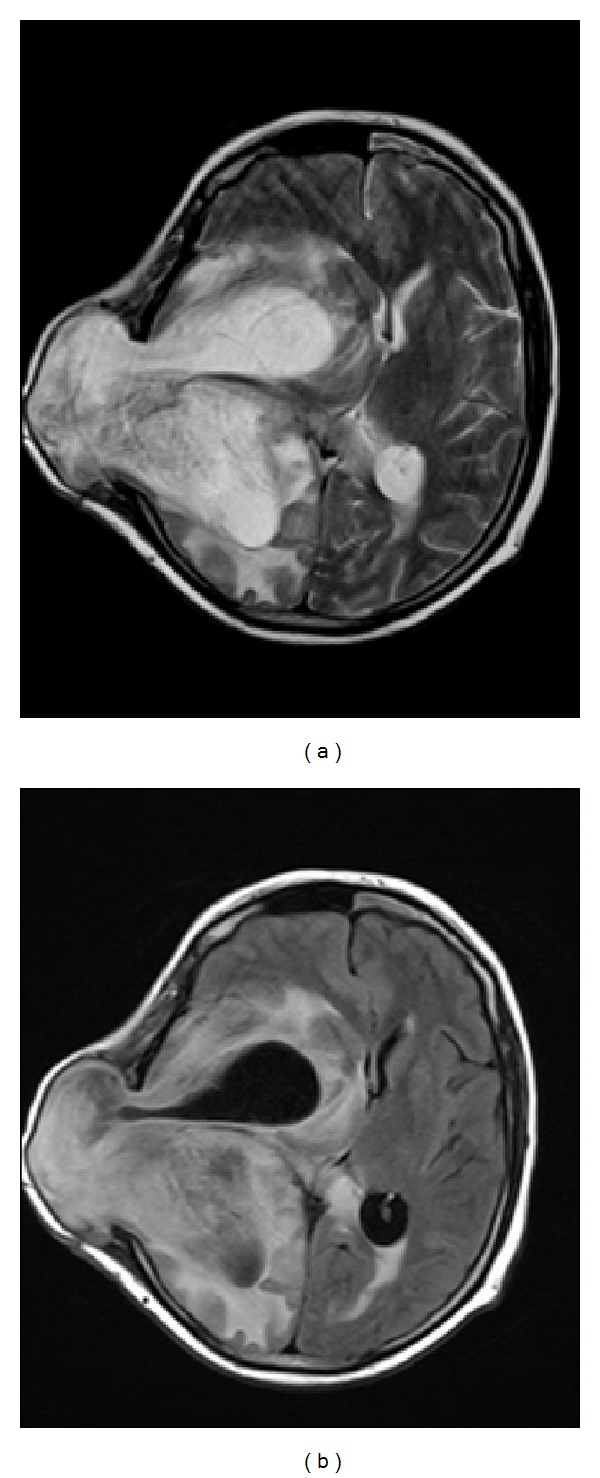
T2 weighted (a) and (b) FLAIR MR images of the lesion before the delivery on axial plane showing a recurrent lesion at the same location. In this figure we see herniated parenchymal tissue from craniectomy defect. There is midline shift about 18 mm in diameter. Mesencephalon and pons retracted to left and there is also uncal herniation.

A 30-year-old woman was admitted to emergency room with the complaint of severe headache at the seventh week of pregnancy. Unenhanced brain magnetic resonance imaging (MRI) revealed a heterogeneous mass lesion at the right temporal lobe (Figures 1(a), 1(b), and 1(c)). Her neurological examination was intact on the admission. Patient was operated on after approval of physicians of obstetricians and gynecologists. Pathological diagnosis was reported as gliosarcoma (Figures 2(a), 2(b), 2(c), and 2(d)). Family was advised to terminate the pregnancy. Radiotherapy and chemotherapy were planned; however, family rejected this advice and they decided to continue the pregnancy. At the 24th week of pregnancy patient attended with severe headache and left sided hemiparesis. Unenhanced brain MRI revealed a recurring lesion at the same location with prominent peripheral edema and minimal midline shift (Figures 3(a) and 3(b)). Patient was operated on again. Bone flap was embedded in abdomen. Histopathological diagnosis was again consistent with gliosarcoma. Hemiparesis was improved in the early period after surgery. 20 days after surgery (at the 27th week of pregnancy) radiotherapy was given. At the 37th week of the pregnancy, delivery with cesarean section (C/S) was planned. Before C/S, control brain MRI revealed the massive progression of the tumor 4 (Figures 4(a) and 4(b)). During C/S, also surgery for recurring lesion in the brain was performed.

Oncology consultation was requested. Due to very low Karnofsky performance scale and low survival expectation of the patient and the possibility of chemotherapeutic agents increasing the morbidity, patient was not given any chemotherapeutic agent. Patient died 2 months after delivery.

## 3. Discussion

Although primary gliosarcoma was classified as a variant of glioblastoma multiforme by WHO (World Health Organization) in 2000 [5], the current accepted definition is that primary gliosarcoma is a well-circumscribed lesion composed of clearly identifiable gliomatous and metaplastic mesenchymal components [6, 7]. This pathology mostly affects patients in the fifth to seventh decade of life. Men were more commonly affected [7]. Our case was differing from the established literature data with two aspects. First one of them is the age. The second one is the gender. Our patient was a 30-year-old woman. We do not have sufficient data about the incidence of gliosarcoma in this age group. Established data in the literature suggest that this pathology is more common in adult males at a ratio of 1.8 to 1.

Primary intracranial tumors are rare in women of reproductive ages (20 to 39 years of age). However, they are the fifth most common cause of death due to cancer. The most common primary tumor is the glial tumors and then meningiomas and acoustic neuromas follow them with decreasing frequency. When pregnant and nonpregnant women of the same age were compared, no difference in incidence of primary brain tumors was detected [8].

The interaction between pregnancy and glial tumors has not been well documented. It has been generally accepted that pregnancy might have a positive impact on glioma growth [9–11]. In fact, pregnancy does not increase the risk of brain tumor; however, it affects the biological behavior of the tumor by multiple factors including hormone, growth factor, and hemodynamic changes [9, 12, 13]. It was reported that increased rate of gliomas during pregnancy might be due to activation of specific receptors by these hormones especially progesterone which enhances cell growth in human gliomas via progesterone receptor- (PR-) B while inhibiting the growth via PR-A. Additionally, secretion of placental growth hormone at the maternal placental interface stimulates secretion of growth factors [9, 12, 13]. Also, pregnancy may change the time of the onset of first signs and it may diverge in the development of symptoms [14]. Peritumoral edema and absolute tumor blood volume may increase during pregnancy due to increased systemic and cerebral blood volume [9]. Additionally, in a recent study, Daras et al. reported the tumor progression and transformation of low grade glial tumors to high-grade gliomas (WHO III and IV) [15].

The current literature about pregnancy and glial tumors is limited to case reports and small case series. In most of these reports, glial tumors were low-grade according to WHO classification. However, cases with glioblastoma multiforme in pregnancy were reported [16, 17]. But to the best of our knowledge, the literature does not contain a gliosarcoma case diagnosed and managed during pregnancy.

In the previous studies, it was reported that congenital abnormality or fetal losses were not observed due to radiotherapy during the advanced gestational age [18]. In our case, we gave radiotherapy 3 weeks after the second operation.

In conclusion, in the management of such cases we need more new information from studies including large number of patients. In particular, data about using radiotherapy and chemotherapy during which trimesters of pregnancy are needed. The management of patients with brain tumors is not dependent of a single department; it is a work of multidisciplines.
